# Structural Insights of the ssDNA Binding Site in the Multifunctional Endonuclease AtBFN2 from *Arabidopsis thaliana*

**DOI:** 10.1371/journal.pone.0105821

**Published:** 2014-08-26

**Authors:** Tsung-Fu Yu, Manuel Maestre-Reyna, Chia-Yun Ko, Tzu-Ping Ko, Yuh-Ju Sun, Tsai-Yun Lin, Jei-Fu Shaw, Andrew H.-J. Wang

**Affiliations:** 1 Institute of Bioinformatics and Structural Biology, National Tsing Hua University, Hsinchu, Taiwan; 2 Institute of Biological Chemistry, Academia Sinica, Taipei, Taiwan; 3 Agricultural Biotechnology Research Center, Academia Sinica, Taipei, Taiwan; 4 Institute of Plant and Microbial Biology, Academia Sinica, Taipei, Taiwan; 5 Department of Biological Science and Technology, I-Shou University, Kaohsiung, Taiwan; Ghent University, Belgium

## Abstract

The multi S1/P1 nuclease AtBFN2 (EC 3.1.30.1) encoded by the *Arabidopsis thaliana* At1g68290 gene is a glycoprotein that digests RNA, ssDNA, and dsDNA. AtBFN2 depends on three zinc ions for cleaving DNA and RNA at 3′-OH to yield 5′-nucleotides. In addition, AtBFN2′s enzymatic activity is strongly glycan dependent. Plant Zn^2+^-dependent endonucleases present a unique fold, and belong to the Phospholipase C (PLC)/P1 nuclease superfamily. In this work, we present the first complete, ligand-free, AtBFN2 crystal structure, along with sulfate, phosphate and ssDNA co-crystal structures. With these, we were able to provide better insight into the glycan structure and possible enzymatic mechanism. In comparison with other nucleases, the AtBFN2/ligand-free and AtBFN2/PO_4_ models suggest a similar, previously proposed, catalytic mechanism. Our data also confirm that the phosphate and vanadate can inhibit the enzyme activity by occupying the active site. More importantly, the AtBFN2/A_5_T structure reveals a novel and conserved secondary binding site, which seems to be important for plant Zn^2+^-dependent endonucleases. Based on these findings, we propose a rational ssDNA binding model, in which the ssDNA wraps itself around the protein and the attached surface glycan, in turn, reinforces the binding complex.

## Introduction

Nucleases catalyze the cleavage of phosphodiester bonds in nucleic acids, and can be classified into DNases and RNases [1]. The multi S1/P1 nucleases have a broad specificity. They can cleave both single-stranded DNA (ssDNA) and RNA, but show a lower affinity for dsDNA. The S1/P1 nuclease class takes its name from P1 nuclease from *Penicillium citrinum* [2] and Nuclease S1 from *Aspergillus oryzae* [3], [4], which are both glycosylated endonucleases, and share 51% sequence identity (68% homology). S1/P1 endonucleases are also present in plants, and are involved in programmed cell death (PCD) via hydrolysis of genomic DNA [5]. Plant S1/P1 endonucleases are divided into two broad types according to their metal ion cofactor: Ca^2+^-dependent and Zn^2+^-dependent endonucleases (EC 3.1.30.1) [6]. Ca^2+^-dependent endonucleases prefer ssDNA over RNA at neutral pH, and may be involved in plant immunity [7]. Acidic pH leads to an increase in activity of Zn^2+^-dependent endonucleases including *Arabidopsis thaliana* multifunctional Nuclease 1 (AtBFN1 or ENDO1) [8], [9], AtBFN2 [10], BEN1 [11], CEL1 [12], ZEN1 [13], ABN1 and HBN1 [14]. N-Glycosylation patterns are highly conserved within the plant S1/P1 endonucleases [15], although these sites are not consistent with those in P1 nuclease [16].

AtBFN2 (also called ENDO2) is a 34.5 kDa glycoprotein (the predicted mass for protein component alone is 30.5 kDa), encoded by the *Arabidopsis thaliana* At1g68290 gene. AtBFN2 follows the standard glycosylation pattern of plant S1/P1 endonucleases, with three glycosylation sites (Asn91, Asn110, and Asn184). While the P1 nuclease has a higher specificity for RNA over ssDNA, in a previous study we showed that the opposite is true for AtBFN2 [10]. In previous studies we have further shown that the enzymatic activity is strongly glycan dependent, which was fully inhibited by complete de-glycosylation, after treatment with PNGase F [10].

Structurally, Zn^2+^-dependent endonucleases present a unique fold, and are classified into the P1/S1 nuclease family (pfam ID PF02265) [17], itself part of the Phospholipase C (PLC)/P1 nuclease superfamily (pfam ID CL0368) [18]. The active site within the P1/S1 nuclease family is very well conserved [19], [20]. It contains nine zinc cluster interacting amino acids (1 tryptophan; 3 aspartic acids and 5 histidines) [15]; the conservation of this metal cluster extends into the PC-PLC/P1 endonuclease superfamily despite low sequence identity [21].

Despite extensive efforts to understand how Zn^2+^-dependent endonuclease bind and digest ssDNA [16], [22], it is still not clear how, or even whether, these endonucleases unwind and cleave dsDNA. Further, the mechanism by which endonucleases specifically interact with ssDNA remains obscure. All co-crystal structures show partially digested DNA in the active site [16], or crystalline artifacts outside of it [17]. Additionally, though it has been previously suggested that glycosylation is relevant for protein structural integrity [10], a role for glycans in substrate binding or modulation of active site shape may also be important.

To address these open questions, we extended our previous studies based on AtBFN2 [16]. We improved the resolution of our earlier sulfate co-crystal [16] from 1.76 Å to 1.22 Å, thus yielding more insight into the glycan structure. We obtained a ligand-free AtBFN2 structure, as well as a phosphate co-crystal structure in a new unit cell with space group *P*1. We also found that phosphate and its transition state analog vanadate were both capable of inhibiting enzyme activity. Finally, we obtained a detailed ssDNA•AtBFN2 co-crystal structure by soaking the crystals with a thiophosphorylated ssDNA analog. In this structure a large secondary ssDNA binding site was observed. Based on these findings, we propose a rational model for ssDNA•AtBFN2 binding, in which the ssDNA wraps itself around the protein and the attached surface glycan, in turn, reinforces the complex, acting not unlike an elastic band.

## Materials and Methods

### Cloning, overexpression, mutagenesis and purification of AtBFN2

The ENDO2 cDNA was amplified by PCR with primer pairs B2-11F and B2-12R to include a C-terminal 6 x His-tag (Table S1, file S1). The ENDO2-His-tag was cloned into pBI121 binary vector to form CaMV35SP::ENDO2-hisOE, and introduced into *Agrobacterium tumefaciens* strain GV3101::pMP90 by electroporation. Arabidopsis plant transformation was performed with the vacuum infiltration method (http://transplant.sinica.edu.tw/english/protocol/trans/1.htm). Seeds were surface-sterilized in 1% NaOCl and 0.05% Tween-20 solution for 15 minutes. After washing with sterile distilled water, seeds were plated in kanamycin-selection medium (1/2 Murashige-Skoog medium containing 0.11 g l^−1^ B5 vitamins, 1% sucrose, 50 mg l^−1^ kanamycin, pH 5.7 and 0.8% Phyto agar). Seedlings were grown under long-day conditions (16 h light/8 h dark) at 22±1°C with light irradiance of 150 mol m^−2 ^s^−1^ and relative humidity of 55%. Kanamycin-resistant seedlings were planted in soil. After siliques wilting, seeds were collected and stored at 4°C. Two week-old seedlings (homozygous) were harvested to extract the AtBFN2 protein for purification.

AtBFN2 was purified following the method of Ko et al. with minor modifications [10]. Soluble proteins were extracted from transgenic seedlings with 20 mM Tris buffer (pH 7.5) containing an EDTA-free protease inhibitor cocktail (Roche, Germany). After centrifuging at 27000 g for 90 min, the supernatant was loaded into a Q column (Q Sepharose Fast Follow, GE Healthcare, USA), equilibrated and washed with 20 mM Tris buffer. The AtBFN2 was eluted and recovered by the His-wash buffer (20 mM Tris, pH 7.5 with 250 mM NaCl). The fraction was loaded to a Ni Sepharose 6 Fast Follow (GE Healthcare) column equilibrated with His-wash buffer. After washing with His-wash buffer, AtBFN2 was eluted with elution buffer (20 mM Tris, pH 7.5, 250 mM NaCl and imidazole from 0 to 250 mM). The collected fractions (60 to 120 mM imidazole) were applied to 5ml Concanavalin A column (Con A, Sigma, USA) washed with Con A pre-washing solution (1 M NaCl, 5 mM MgCl_2_, 5 mM MnCl_2_, 5 mM CaCl_2_), and then equilibrated with Con A wash buffer (20 mM Tris, pH 7.5, 500 mM NaCl). After washing with Con A wash buffer, the AtBFN2 was eluted with Con A elution buffer containing 5 to 500 mM glucose and α-D-mannopyranoside. The Con A eluted fractions were concentrated with 10 kDa Vivaspain turbo (Sartorius Stedim Biotech, UK) then applied to a Sephacryl S-100 High Resolution (GE Healthcare) column in buffer (20 mM Tris pH 7.5, 50 mM NaCl). Recombinant AtBFN2 protein was detected with 12% SDS-PAGE. Purified AtBFN2 was concentrated with 10 kDa Vivaspain turbo for crystallization.

### Crystallization, data collection, and structure determination

All crystals were obtained using the sitting-drop vapor diffusion method at temperatures ranging from 293 to 298 K. Ligand-free AtBFN2 crystals grew within 5 to 7 days in drops prepared by mixing 1 µl of the reservoir solution (0.1 M Tris pH 8.5, 0.2 M NaOAc, 30% (w/v) PEG 4000) with 1 µl of the protein (6.0 mg/ml). All AtBFN2/SO_4_; AtBFN2/PO_4_ and AtBFN2/A_5_T crystals grew within 3 to 4 weeks in drops prepared by mixing 1 µl of the reservoir solution (0.1 M Hepes, pH 7.5; 0.2 M LiSO4 and 30% (w/v) PEG 3350 for AtBFN2/SO4 and AtBFN2/A5T; 0.1 M Hepes, pH 7.5; 0.2 M Na2HPO4 and 30% (w/v) PEG 3350 for AtBFN2/PO_4_) with 1 µl of the protein (8.5 mg/ml). The AtBFN2/A_5_T co-crystals included thiophosphorylated nucleotides: d[A(s)A(s)A(s)A(s)A(s)T], denoted as A_5_T. AtBFN2/SO_4_ crystals were washed in 0.1 M Hepes, pH 7.5 and 30% (w/v) PEG 3350 for 5 seconds, and then soaked in 0.1 M Hepes, pH 7.5 and 30% (w/v) PEG 3350 with 0.2 mM A5T for 30 minutes. Subsequently, they were flash-cooled to 100 K in a stream of cold nitrogen.

The X-ray diffraction images were collected using the SPXF beamline BL13B1; BL13C1 and BL15A1 of the National Synchrotron Radiation Research Center (NSRRC) in Taiwan, and were then processed using HKL2000 [23]. Prior to use in structure determination, 5% randomly selected reflections were set aside as our Rfree reference [24]. The statistics of data collection are summarized in Table 1.

**Table 1:** Data collection and refinement statistics. Numbers in parentheses are for the highest resolution shells. All positive reflections were used in the refinement.

	AtBFN2/ligand-free	AtBFN2/SO_4_	AtBFN2/PO_4_	AtBFN2/A_5_T
**Data Collection**				
Space group	*P*1	*P*2_1_2_1_2_1_	*P*1	*P*2_1_2_1_2_1_
Unit-cell *a, b, c* (Å)	45.4, 52.7, 61.1	54.1, 66.8, 99.6	45.5, 52.9, 60.8	53.9, 66.2, 98.1
Unit-cell *α, β, γ* (°)	71.3, 78.6, 76.7	90.0, 90.0, 90.0	71.3, 78.6, 76.6	90.0, 90.0, 90.0
Resolution (Å)	50–2.10 (2.18–2.10)	50–1.22 (1.26–1.22)	25–2.0 (2.07–2.00)	50–1.67 (1.74–1.67)
Unique reflections	29732 (2961)	107958 (10692)	34563 (3403)	39430 (3873)
Redundancy	3.9 (3.9)	11.7 (10.5)	2.0 (2.0)	9.9 (9.5)
Completeness (%)	98.0 (97.1)	99.9 (99.9)	96.7 (95.2)	94.8 (95.7)
Average I/σ (I)	14.7 (3.7)	35.3 (3.5)	9.4 (3.3)	28.9 (2.2)
R_merge_ (%)	10.0 (44.8)	5.6 (45.8)	6.9 (26.5)	6.5 (71.0)
**Refinement**				
No. of reflections	28226 (1882)	102490 (7495)	31321 (1659)	36995 (2645)
R_work_ (95% of data)	19.7 (20.8)	11.8 (17.4)	20.2 (27.3)	14.0 (15.5)
R_free_ (5% of data)	26.8 (31.2)	13.3 (18.8)	24.9 (34.8)	19.1 (23.7)
R.m.s.d. bonds (Å)	0.011	0.010	0.010	0.008
R.m.s.d. angles (°)	1.564	1.628	1.563	1.47
Dihedral angles				
Most favored (%)	94.6	97.8	95.67	96.4
Allowed (%)	5.4	2.2	4.33	3.6
Disallowed (%)	0.0	0.0	0.0	0.0
Average B (Å^2^)/atoms				
Protein	26.6/4098	13.8/2148	28.2/4087	25.9/2019
Carbohydrate	41.7/256	24.7/193	38.6/231	42.6/150
Ions & ligands	19.7/6	21.7/28	17.9/16	50.0/71
Water molecules	35.0/457	36.1/493	36.6/426	42.8/324
**PDB ID code**	**4CWM**	**4CXP**	**4CXV**	**4CXO**

The structure of AtBFN2 crystals were solved by using molecular replacement with the PDB ID: 3W52 as a search model [16]. All calculations were carried out by using CNS [25] and CCP4 [26]. The program COOT was employed for model building [27]. The models were further improved by introducing polysaccharides, sulfate ions, water molecules, and nucleotides. Statistics of the refined structure were produced using REFMAC5 [28], [29] and MolProbity [30] and can be found in Table 1. All comparison of the structures were performed using the PyMOL Molecular Graphics System, Version 1.5.0.4 Schrödinger, LLC.

### ssDNA activity assay

ssDNA activity was measured in triplicate according to the method of Ko *et al.* [10]. Purified AtBFN2 protein (0.1 µg) was incubated with 1 mg of heat-denatured ssDNA substrate in 200 µl of various buffers in each 1.5 ml Eppendorf tube at 37°C, 10 minutes. All assays were performed in 100mM Tris pH 7 buffer, to which different putative inhibitors were added (100 mM Na_2_SO_4_; 100 mM LiCl; 100 mM Li_2_SO_4_; 1 mM and 10 mM Na_3_VO_4_; 1 mM and 10 mM and 100 mM NaH_2_PO_4_). In order to stop the reaction, we added 50 µl of cold 1 M HCl, followed by a 10 minute incubation period on ice. After centrifugation at 16000 g for 30 minutes, the supernatant was measured at 260 nm to determine the amount of acid-soluble DNA using NaroDrop 2000 (Thermo, USA).

## Results and Discussion

### Extended N-glycan structures

**Figure 1:**
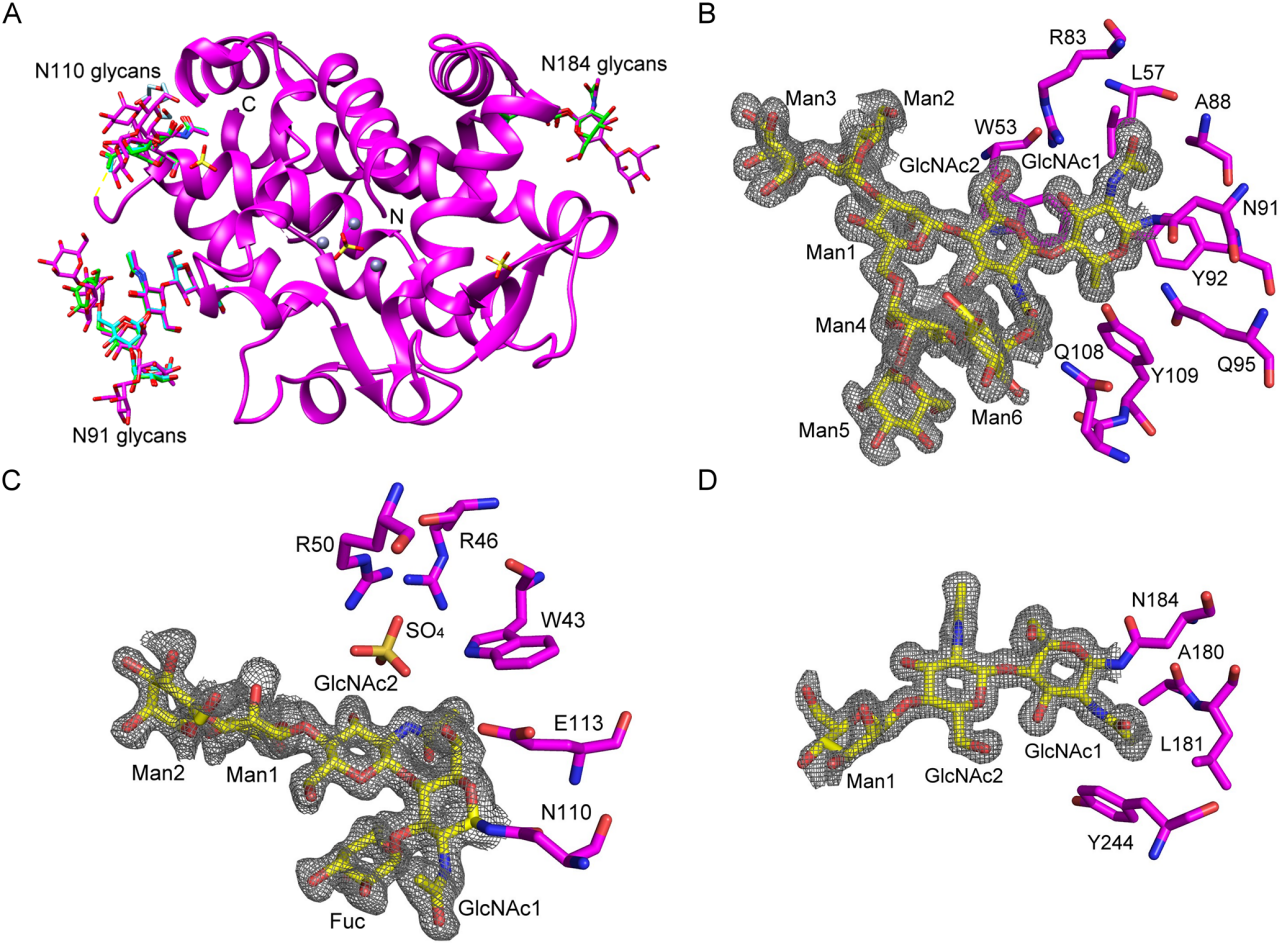
Overall structure of AtBFN2 and extended glycan structures. A: In the ribbons diagram, AtBFN2•SO_4_ (3W52, green) [16], Ligand-free AtBFN2_A chain (4CWM, cyan) and AtBFN2/SO_4_ (4CXP, magenta) structures are superimposed (3W52 RMSD over 224 common Cα of 0.2 for chains A in 4CWM, and over 209 common Cα of 0.078 in 4CXP). Zinc and sulfate ions are shown as gray spheres and yellow sticks. The N-glycan structure is labeled for three N-glycosylation sites, 91, 110 and 184 in the same color as their respective main chains (only 4CXP is shown). Protein termini are labeled N and C, whereas the disordered region of residues 101–106 is indicated by yellow dots. B–D: Detailed 4CXP N-glycan structures and electron density maps. Glycans are shown as sticks and labeled, and the omit map contoured at 0.6σ (gray) for glycans. Protein residues interacting with the glycans are labeled and shown as sticks. B: N-glycosylation site at Asn91; C: N-glycosylation site at Asn110 and D: N-glycosylation site at Asn184.

The protein portion of the sulfate co-crystal structure (4CXP) presented in this work is in very good agreement with our previously published AtBFN2•SO_4_ co-crystal structure (3W52), which showed the overall fold of this endonuclease (Fig. 1A) [16]. The improved diffraction quality of 4XCP, however, allowed us to refine the structure to include almost all putative glycans, as described by Ko *et al.* [10], with only six moieties still missing (Fig. 1). As with the 3W52, 4XCP shows three N-glycosilation sites, at Asn91, Asn110, and Asn184.

The glycan bound to Asn91 is especially well defined and we can discern both N-acetylglucosamines (GlcNAc), and a further six mannose moieties (Man) (Fig. 1B). Much like in 3W52, GlcNAc1 interacts extensively with the polypeptide, forming hydrogen bonds with Arg83, Tyr92, and Gln95. Further, the methyl group of GlcNAc1 is located within a small aliphatic pocket formed by Leu57, and Ala88. GlcNAc2, on the other hand, interacts via π-ring stacking with Trp53. Man 1–5 do not interact with the protein. Man6, on the other hand, folds backwards into the protein, and interacts with the side chain of Gln108 and with the carbonyl group of GlcNAc2 (Fig. 1B).

While we were able to refine only one additional mannose moiety on the glycan bound to Asn184 (Fig. 1D), we obtained a much more detailed structure for the one attached to Asn110. It was thus possible to observe two more Man residues, which extend towards the solvent, and do not interact directly with the protein (Fig. 1C). A fucose moiety (Fuc) is attached to GlcNAc1 via an α1–3 glycoside bond, but it also makes no direct contacts with the protein.

Interestingly, GlcNAc1 at Asn110 interacts specifically with a single sulfate molecule, which itself interacts with Arg46 and Arg50, and with the aromatic nitrogen of Trp43. The sulfate binding pocket seems well prepared to bind ligands which are both negatively charged and contain de-localized π-orbitals. It is tempting to think that this might be a possible accessory binding site for ssDNA.

### Structure and activity of the active Zn cluster

**Figure 2:**
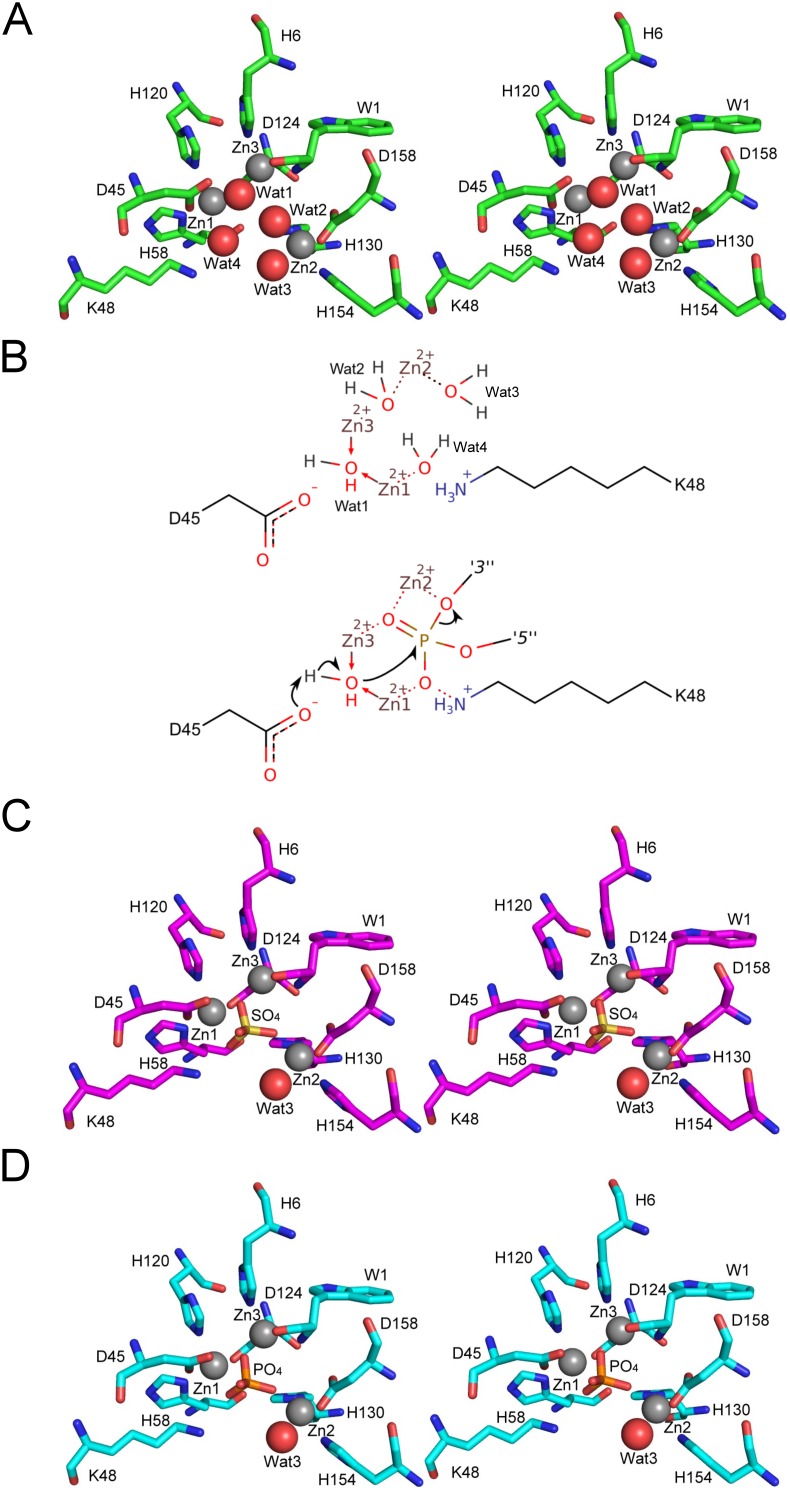
The coordination spheres of the tri-metallic Zn cluster with different ligands. The tri-metallic zinc cluster, shown as gray spheres in all stereo figures, is embedded in a central pocket of the enzyme. A: The ligand-free AtBFN2 (4CWM, green) active site structure shows four water molecules bound separately to the Zn cluster. Water one (Wat1) bridges the gap between Zn1 and Zn3, whereas waters two, three and four (Wat2, Wat3, and Wat4) exclusively bind a single Zn atom each. B: Schematic view of the binding pocket (top), and proposed activated nucleophile mechanism (bottom). The covalent bonds are depicted by solid lines and the non-covalent interactions by dashed lines. The arrows denote the paths of electrons during the catalysis. The phosphate is connected to the preceding R_1_ and the succeeding R_2_ nucleosides by the O3′ and the O5′ atoms, respectively. When Wat1 is activated by D45, it attacks the phosphate, which is being stabilized by the other Zn atoms, and K48. As a result, the bond with the 3′ nucleoside is broken. C: AtBFN2/SO_4_ (4CXP, magenta) reveal a sulfate anion bound within the active site, displacing Wat1, Wat2 and Wat4. D: AtBFN2/PO_4_ (4CXV, cyan), with a phosphate anion in the same position as the sulfate was in C.

In addition to our improved AtBFN2•SO_4_ co-crystal structure, we were also able to obtain a new, ligand-free, structure, which was solved from crystals belonging to space-group *P* 1 (Fig. 1A). In this structure, we found that four water molecules coordinate the tri-metallic cluster (Fig. 2A). Water one (Wat1) bridges the gap between Zn1 and Zn3, while waters two and three (Wat2, Wat3) exclusively bind Zn2. Finally, water four (Wat4) coordinates Zn1. Wat1 is in the predicted required position for an activated nucleophile [16], further strengthening our earlier proposal for a reaction mechanism (Fig. 2B). In the SO_4_ co-crystal structure, however, only Wat3 remains (Fig. 2C).

Accordingly, crystals grown in conditions where lithium sulfate was substituted by sodium phosphate, have a phosphate anion displacing all coordinating waters, except Wat3 (Fig. 2D).

**Figure 3:**
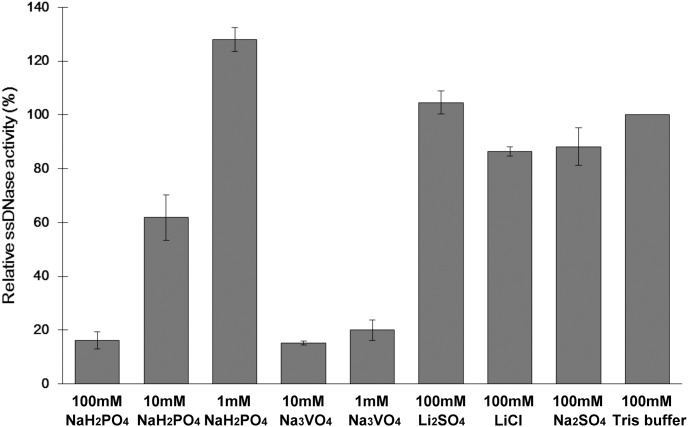
The inhibitory effect of phosphate, and the phosphate analog on AtBFN2. Normalized AtBFN2 nuclease activity was assayed with ssDNA in the presence of various additives. Both phosphate and vanadate inhibit AtBFN2, while the others do not. Normalization was calculated against the standard conditions (100 mM Tris buffer) corresponding to 277.91 ng/µl digested ssDNA.

Endonuclease activity inhibition assays confirmed Wat1 displacement by phosphate and/or sulfate anions. Indeed, phosphate inhibited DNA degradation by 40% at a 10 mM concentration, and by almost 85% at a 100 mM concentration (Fig. 3). Sulfate, however, did not affect enzymatic activity at the tested concentrations, suggesting a much lower affinity for sulfate than for phosphate, as had been hinted by previous experiments with PC-PLC [31]. To confirm the effects of phosphate we tested the well-known phosphatase inhibitor vanadate, which is also a phosphate analog [32] (Fig. 3). As expected, vanadate affected endonuclease activity at lower concentrations than phosphate, achieving 80% inhibition at just 1 mM concentration.

While these results clearly indicate that phosphate, and molecules similar to phosphate, have an inhibitory effect on AtBFN2, it is still not clear what the mechanism of inhibition is. Since phosphodiester bonds are the main substrate of AtBFN2, it is possible that phosphate acts via competitive inhibition. On the other hand, in all published mechanisms for endonuclease activity, the nucleic acid phosphate backbone displaces Wat2, Wat3 and Wat4, but not Wat1, which is necessary for catalysis [16], [17]. Wat1, on the other hand, is only displaced by the cleaved product.

Furthermore, in Endo IV, an unrelated endonuclease which nevertheless contains an extremely similar Zn tri-metallic binding pocket [33], mutagenesis studies produced a variant with a phosphate molecule in the binding pocket, resulting in an inactive protein. Garcin *et al.* concluded that the phosphate coordination in the mutant was not functional, and could be one of the factors leading to total protein inactivation, mimicking the cleaved product [33]. This all leads us to question the competitive inhibition hypothesis. We propose, therefore, that phosphate inhibition might not be competitive, as it would not interfere with ssDNA binding, instead acting by disrupting the active site native coordination sphere.

### The ssDNA•AtBFN2 complex structure reveals a secondary, glycan dependent ssDNA binding site

**Figure 4:**
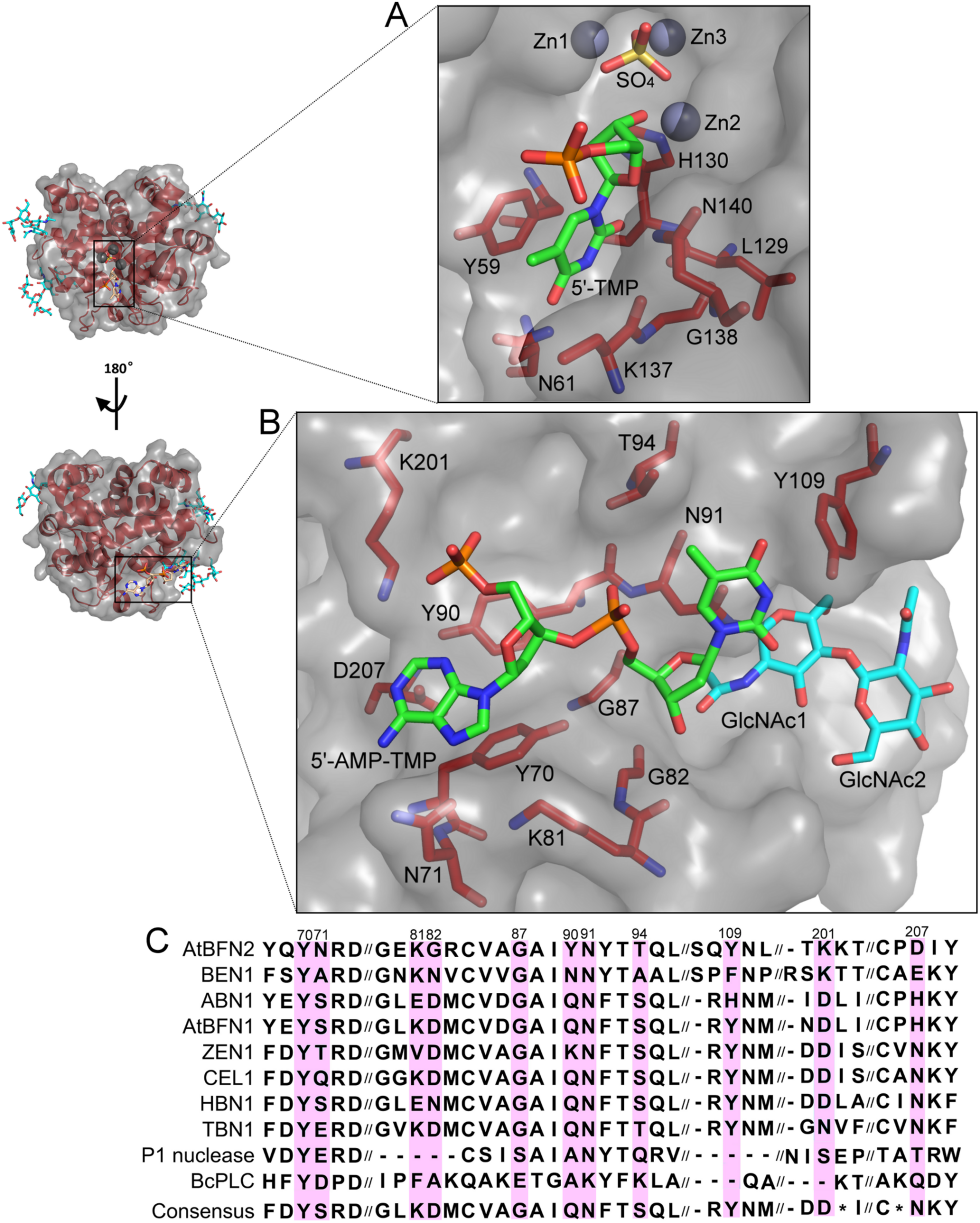
ssDNA binding sites. Two nucleotide binding sites were revealed in the AtBFN2/A_5_T complex structure. A: nucleotide binding site in the active site with 5′-TMP and interacting protein residues. B: secondary nucleotide binding site with dinucleotide 5′-AMP-TMP, interacting protein residues and glycans. C: Alignment of the conserved residues in the secondary binding site.

By soaking our crystals in a 0.2 mM solution of the thio-ssDNA analog A_5_T (sequence: d[A(s)A(s)A(s)A(s)A(s)T]), we managed to obtain the first ssDNA**•**AtBFN2 co-crystal structure (4CXO, Table 1). Here we observed two distinct binding pockets. A single deoxythymidine monophosphate residue was bound to the active site, in much the same way as has been described for P1 nuclease [17]. We were unable to refine any further nucleotides at this site, indicating that the rest of the hexanucleotide was probably highly flexible. The thymine moiety was stacked against Tyr59 and lodged within a pocket comprising the peptide backbones of His130, Lys137, and Gly138, and the side-chains of Tyr59, Asn61, and Leu129 (Fig. 4A). Asn61 bound the thymine carbonyl oxygen 4 via a hydrogen bond. The deoxyribose was bound to Asn140 at the ring oxygen, while the 3′-end was tightly coordinated to Zn2, displacing the Wat3, which was present in the other three structures. Under these conditions, we also found a single sulfate anion within the binding pocket, which had displaced the other waters, including catalytically relevant Wat1.

The secondary binding site is completely novel, and much larger than the active site pocket. In it, elements from both the protein and the N-glycan at Asn91 participate in the binding of a dinucleotide composed of one adenosine, and one thymidine (Fig. 4B). As with the active site, a large part of the ssDNA molecule could not be refined. However, since thymidine was present in both binding sites, it follows that the two binding sites are occupied by distinct molecules. At the 5′-end of the single stranded dinucleotide, the adenosine is bound by an extensive network of hydrogen bonds and hydrophobic interactions, with Tyr70 stacking with the purine moiety. Further, the amide belonging to Asn71 coordinates the aromatic nitrogen 7 and the primary amine group on the adenine ring. The deoxyribose portion of the adenosine interacts via a π-ring interaction with Tyr90, while the phosphate backbone attracts the positive side-chain of Lys201.

The thymidine, on the other hand, is bound within a relatively hydrophobic pocket lined by Gly87 at α-helix 7, with glycosylation site 91 in the direct vicinity. Indeed, GlcNAc1 at Asn91 interacts via its acetyl group with the 3′-end of the thymidine deoxyribose, which also interacts with the backbone of Lys81. At the same time, the Asn91 side-chain, and the GlcNAc1 ring stack the thymine moiety. Finally, a hydrophobic contact between Tyr109 and the thymine aromatic ring further stabilizes the glycoprotein-N-glycan-nucleotide complex.

### A wrapped ssDNA binding model

The secondary binding site possibly reveals a new and crucial role for glycosylation at Asn91, since GlcNAc1 is an important partner in binding the dinucleotide. A reasonable hypothesis would be that a longer ssDNA strand would be capable of interaction with further glycans. Thus, we propose that N-glycosylation at Asn91 has not only a role in maintaining structural integrity, but also in substrate binding.

**Figure 5:**
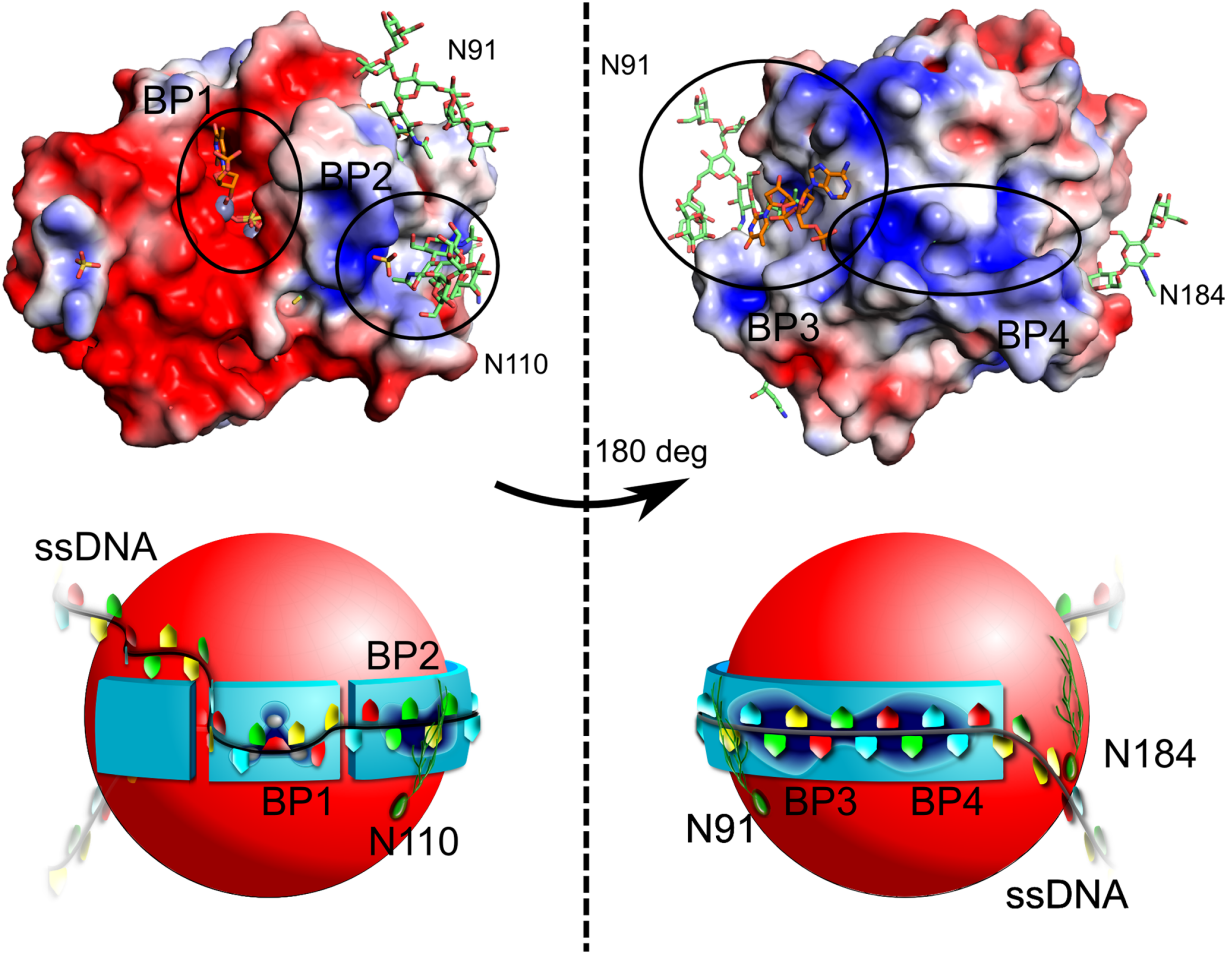
The ssDNA binding pockets and wrapped ssDNA binding model. Top: DelPhi electrostatic surface ranging from -3 (deep red) to 3 (deep blue). Zn is colored gray, and was not used in the electrostatic potential calculations. The model was generated by combining the well-defined glycan structures of 4CXP (green) with the ssDNA ligands of 4CXO (orange). The protein is presented from the active site side, on the left, and rotated by 180 degrees, on the right. Bottom: Schematic representation of the above model. In total, we identified two experimental binding pockets (BP1 and BP3), and we propose two more (BP2 and BP4). BP1 is the active site, in which the ssDNA hydrolysis takes place. BP2 is lined by Trp43, Arg46, Arg50, and GlcNAc1 from N-glycan at position 110. BP3 is the large secondary ssDNA binding site, which was determined in our crystal structure. It is described in greater detail in Fig. 4 and is well conserved in our alignment. BP4 is a positive charge-rich extension of BP3, which in addition contains three tryptophans and one tyrosine (Fig. S2). We propose that the ssDNA will wrap itself around the AtBFN2 molecule via BP2, BP3, and BP4, with further stabilization provided by the N-glycans (green filaments in our figure). The reaction will then take place in BP1.

While it was only possible to observe two nucleotides from our short DNA sequence within the secondary binding pocket, the relative position of these, in comparison with the adenosine in the active site, is suggestive of a long, snaking DNA molecule wrapped around the surface of AtBFN2 (Fig. 5). Based on DelPhi electrostatic calculations [34], we propose that there are two further ssDNA binding pockets (pockets two and four in Fig. 5). Pocket two is occupied in the sulfate co-crystal structure by sulfate (Fig. 1A), suggesting that it should be possible for the ssDNA backbone to be bound in a similar fashion. On the other hand, pocket four, an extension of pocket three, is positively charged, and contains several aromatic residues (Figure 5, and Figure S1 in File S1).

The physiological role of the Tyr site on the P1 nuclease (Tyr144 and Tyr155, Figure S1 in File S1) has not yet been confirmed, and there is still doubt as to its relevance outside the crystal environment, since the key amino-acids are poorly conserved [17]. Therefore we sought to evaluate the degree of conservation, in comparison to the Tyr site, of our experimental and proposed secondary binding sites by performing an alignment based on several plant Zn^2+^-dependent nucleases, and the more distantly related P1 nuclease, and BcPLC (Fig. 4C and Figure S1 in File S1). The majority of the amino-acids involved in the experimental secondary binding site (pocket three) are well conserved within the plant Zn^2+^-dependent nucleases, and the ones with a lesser degree of conservation are replaced by residues capable of similar interactions. For example, Asn71, was conservatively replaced by serine in the consensus sequence (Fig. 4C). On the other hand, Lys201 is most often replaced by glutamate, which is incapable of interacting with the phosphate backbone. However, glutamate side-chains are highly flexible, resulting in low repulsion against the nucleic acid binding. Additionally, there may be alternative pathways by which the binding pocket may attain a similar geometry. For example, the tomato multifunctional endonuclease TBN1 presents a Gln instead of Tyr at position 90 (Fig. 4C). The TBN1 structure (3SNG) [22] also comprises an N-terminal extension (lacking in AtBFN2) of α helix 11 with an exposed phenylalanine. Thus, it is possible that the side-chains of Gln and Phe are able to structurally compensate for the absence of Tyr90 (Figure S2 in File S1). Furthermore, the computed binding sites (pockets two and four) exhibit high sequence similarity, with conserved amino acid substitutions, e.g. Arg to Lys (Figure S1 in File S1). Considering the high levels of sequence and structure conservation in the proposed ssDNA secondary binding sites, it is highly likely that they have some biological significance in plant Zn^2+^-dependent endonucleases. Since, however, their levels of sequence and structure conservation in AtBFN2, and P1 nuclease are low, and since both exhibit different specificities (weaker ssDNA binding to P1 nuclease) the DNA binding mechanism for distantly related endonucleases might differ considerably.

## Conclusion

In this work, we presented structural and functional data confirming that phosphate and vanadate act as inhibitors of AtBFN2 by occupying the active site, and possibly displacing catalytic water. We also obtained high resolution structures with well defined N-glycans at positions 91, 110, and 184 of the protein. Our most important finding, however, was the strong evidence for a secondary ssDNA binding site, for which the N-glycan at position 91 might be crucial. This binding site seems to be important within plant Zn^2+^-dependent and/or ssDNA digesting endonucleases, yet might not have similar relevance for other organisms.

## Supporting Information

File S1 **Supplementary materials file.** This file contains Figures S1 and S2, along with Table S1.
(PDF)
