# Supplementary material for: Structural Insights of the ssDNA Binding Site in the Multifunctional Endonuclease AtBFN2 from Arabidopsis thaliana
Source: PLoS One. 2014 Aug 26;9(8):e105821. doi: 10.1371/journal.pone.0105821 (PMC4144908; doi:10.1371/journal.pone.0105821)
Supplement: File S1 — Supplementary materials file. This file contains Figures S1 and S2, along with Table S1. (PDF) [file pone.0105821.s001.pdf]

May 5, 2014

PLOS ONE

Title: Structural insights of the ssDNA binding site in the bifunctional endonuclease AtBFN2 from *Arabidopsis thaliana*

Supplementary materials

Tsung-Fu Yu [a,b]†, Manuel Maestre-Reyna [b]†, Chia-Yun Ko [c], Tzu-Ping Ko [b] , Yuh-Ju Sun [a], Tsai-Yun Lin [a], Jei-Fu Shaw [c,d]*, and Andrew H.-J. Wang [a,b,c]*

[a] Institute of Bioinformatics and Structural Biology, National Tsing Hua University, Hsinchu 30013, Taiwan

[b] Institute of Biological Chemistry, Academia Sinica, Taipei 11529, Taiwan

[c] Agricultural Biotechnology Research Center, Academia Sinica, Taipei 11529, Taiwan

[d] Department of Biological Science and Technology, I-Shou University, Kaohsiung 84001, Taiwan

†TFY and MMR contributed equally to this work.

*To whom correspondence should be addressed:

JFS: shawjf@isu.edu.tw

AHJW: ahjwang@gate.sinica.edu.tw

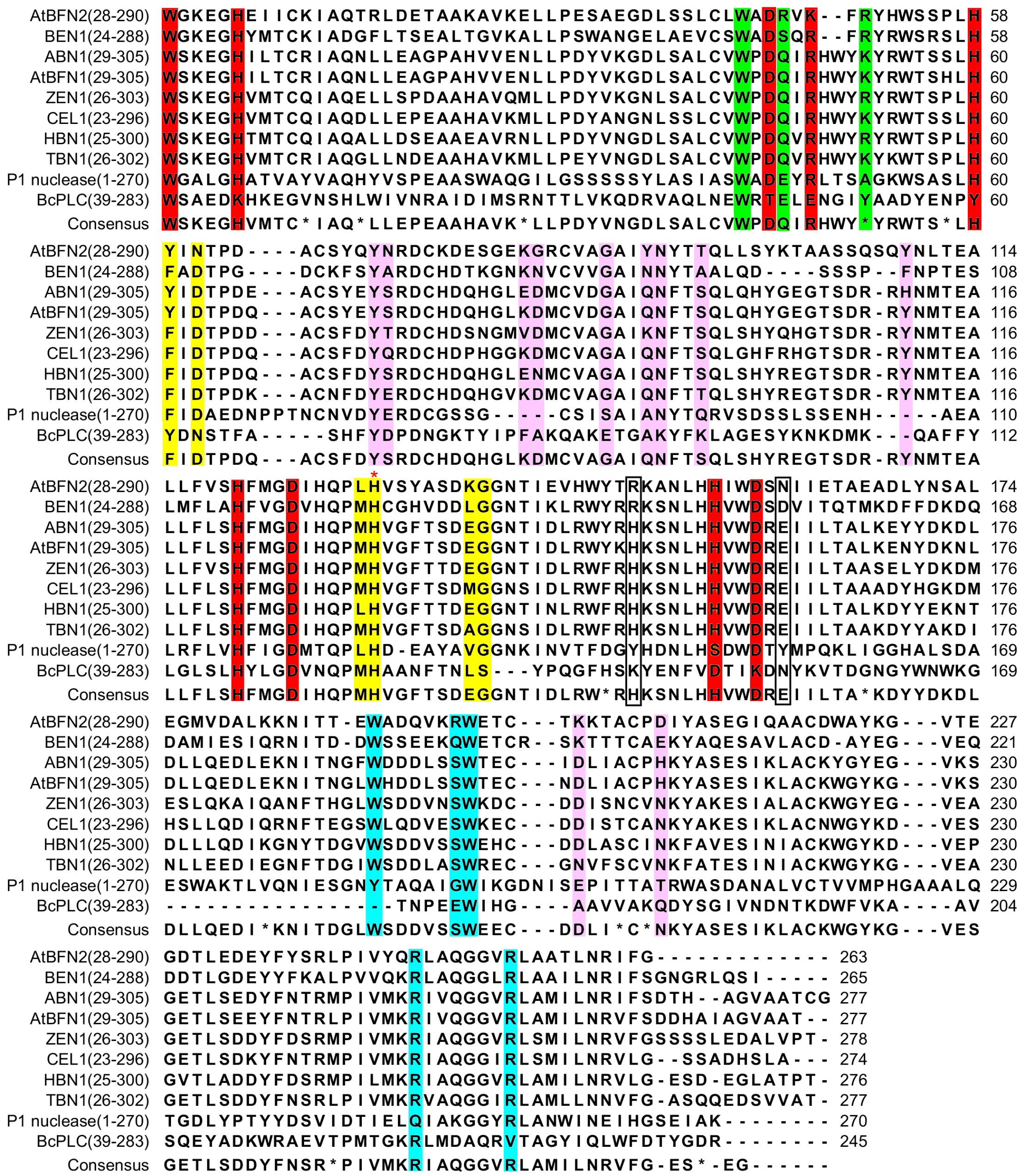

Figure S1. Alignment of S1/P1-type nucleases. Amino acid sequences of AtBFN1 (UnitProt: Q9SXA6), AtBFN2 (Q9C9G4) from *Arabidopsis thaliana*, BEN1 (O81958) from *Hordeum vulgare*, ABN1 (E3PQH5) from *Fourraea alpine*, ZEN1 (O80326) from *Zinnia elegans*, CEL1 (Q9LL59) from *Apium graveolens*, HBN1 (B4ERM5) from *Humulus lupulus*, TBN1 (Q0KFV0) from *Solanum lycopersicum* ,

P1 nuclease (P24289) from *Penicillium citrinum* and BcPLC (P09598) from *Bacillus cereus* were aligned using the ClustalW program. Binding pocket 1 (yellow), pocket2 (green), pocket3 (cyan) and pocket4 (pink) are labeled. The tri-metallic zinc active site is labeled in red and (*). Finally, the highly variable Tyr-site amino-acids are labeled with black outlines.

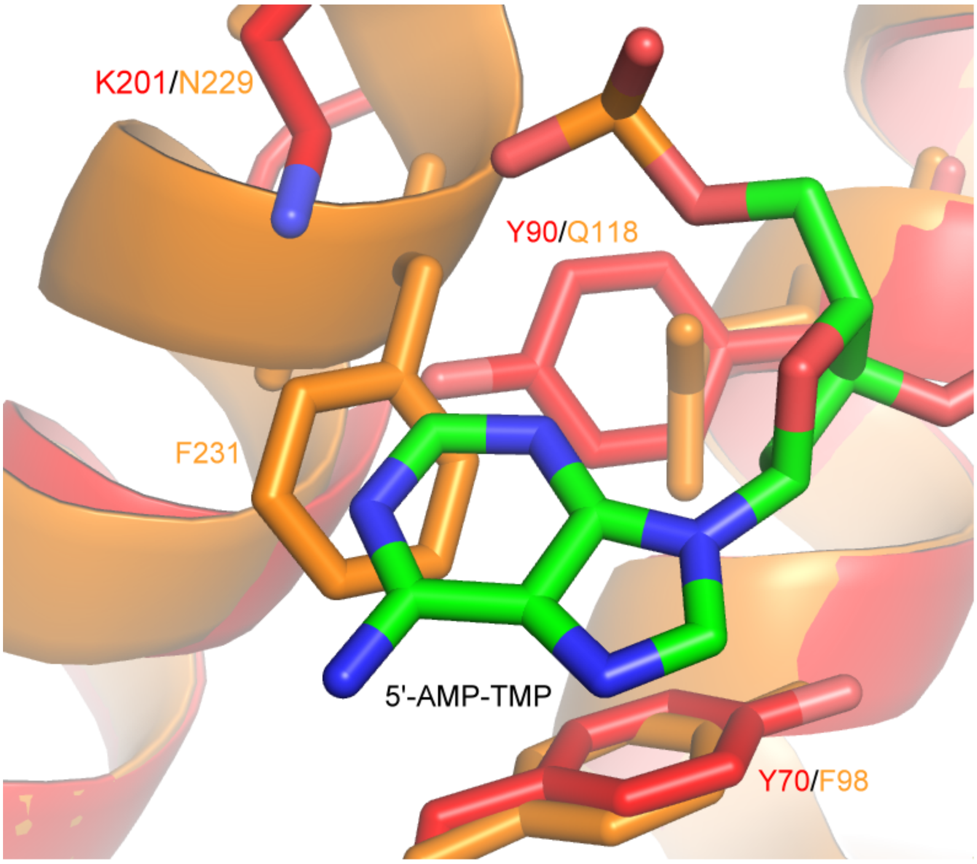

Figure S2. Comparison of secondary binding site between AtBFN2/A5T (red) and TBN1 (3SNG, orange) with conserved protein residues. Tyr90 in AtBFN2 is replaced by a glutamine in TBN1. However, an extension of α helix 11 in TBN1, which AtBFN2 is missing, supplies Phe231.

Table S1. Primers used for PCR-amplification of constructs applied in this study.

For the overexpression construct of ENDO2	
**Sequences of primer pairs (5ㅈ →3ㅈ)**	**Enzyme site**
**B2-11F: CGGGATCCATGGCAAACCAA**	**BamHI**
**B2-12R: CGAGCTCTCAGTGGTGGTGGTGG TGGTGACCGAAAATCCT**	**SacI**
